# One-year outcome of brain injured patients undergoing early neurological rehabilitation: a prospective observational study

**DOI:** 10.1186/s12883-022-02549-w

**Published:** 2022-01-17

**Authors:** Melanie Boltzmann, Simone B. Schmidt, Christoph Gutenbrunner, Joachim K. Krauss, Günter U. Höglinger, Jens D. Rollnik

**Affiliations:** 1 BDH-Clinic Hessisch Oldendorf, Institute for Neurorehabilitation Research, Associated Institute of Hannover Medical School, Hessisch Oldendorf, Germany; 2 Department of Rehabilitation Medicine, Hannover Medical School, Hannover, Germany; 3 Department of Neurosurgery, Hannover Medical School, Hannover, Germany; 4 Department of Neurology, Hannover Medical School, Hannover, Germany

**Keywords:** Neurological rehabilitation, Brain injured patients, Long-term prognosis, Glasgow outcome scale-extended, Coma recovery scale-revised

## Abstract

**Background:**

The present study intended to analyze the outcome of patients with severe brain injury one-year after discharge from early rehabilitation.

**Methods:**

Early neurological rehabilitation patients admitted to intensive or intermediate care units and discharged between June 2018 and May 2020 were screened for eligibility. The level of consciousness was evaluated using the Coma Recovery Scale-Revised (CRS-R) upon admission and at discharge. At one-year follow-up, the outcome was assessed with the Glasgow Outcome Scale-extended (GOSE). Demographical and clinical data collected during inpatient rehabilitation were used to predict the outcome 1 year after discharge.

**Results:**

Two hundred sixty-four patients (174 males, 90 females) with a median age of 62 years (IQR = 51–75) and a median duration of their disease of 18 days (IQR = 12–28) were included in the study. At follow-up, the mortality rate was 27% (*n* = 71). Age and discharge CRS-R total score were independent predictors in a Cox proportional hazards model with death (yes/no) as the dependent variable. According to the GOSE interviews, most patients were either dead (*n* = 71; 27%), in a vegetative state (*n* = 28; 11%) or had a severe disability (*n* = 124; 47%), whereas only a few patients showed a moderate disability (*n* = 18; 7%) or a good recovery (*n* = 23; 9%) 1 year after discharge. Age, non-traumatic etiology, discharge CRS-R total score and length of stay independently predicted whether the outcome was good or poor at follow-up.

**Conclusion:**

Age was an important predictor for outcome at one-year follow-up, which might be due to altered brain plasticity and more comorbidities in elderly subjects. In addition, the present study demonstrated that the CRS-R total score at discharge might be more important for the prediction of one-year outcome than the initial assessment upon admission.

## Background

The number of survivors of severe brain injury has increased over the past 20 years due to advances in emergency medicine, intensive care medicine, and neurosurgical procedures [1]. The acute-care treatment of these patients is often followed by inpatient neurological rehabilitation. Although the condition has been largely stabilized at this point of treatment, most patients are still in need of intensive care, including mechanical ventilation and monitoring of vital parameters. For the clinical management of critically ill patients, information about their prognoses is crucial. Physicians for example need reliable prognostic information to decide upon treatment options [2].

The large heterogeneity of this group, however, makes it challenging to predict the outcome of individual patients. One relevant aspect is the level of consciousness. Patients admitted to neurological rehabilitation are frequently still in an altered state of consciousness, including the unresponsive wakefulness syndrome (UWS; patients show no behavioral signs of self-related or environmental awareness) and the minimally conscious state (MCS; patients show inconsistent but reproducible signs of awareness) [3, 4]. Patients who are in MCS 1 month post-onset are more likely to recover within the first year than patients in UWS [2, 5]. Within both categories, patients with traumatic brain injuries have a better prognosis than patients with non-traumatic brain injuries [2].

In general, prognostic evaluations start as soon as patients are admitted to acute-care hospitals. Several studies, however, suggest that a considerable proportion of critically ill patients recover later, in particular during post-acute inpatient rehabilitation [6–8] or even years after the injury [9–11]. In a recent study investigating critically ill patients receiving post-acute rehabilitation, outcome at discharge was independently predicted by age, initial CRS-R score and gains in CRS-R score after 4 weeks [12]. The present study aims to extend previous findings by following up the outcome of these patients 1 year after discharge. Therefore, prognostic information collected upon admission and at discharge were used to predict the outcome at one-year follow up.

## Methods

**Fig. 1:**
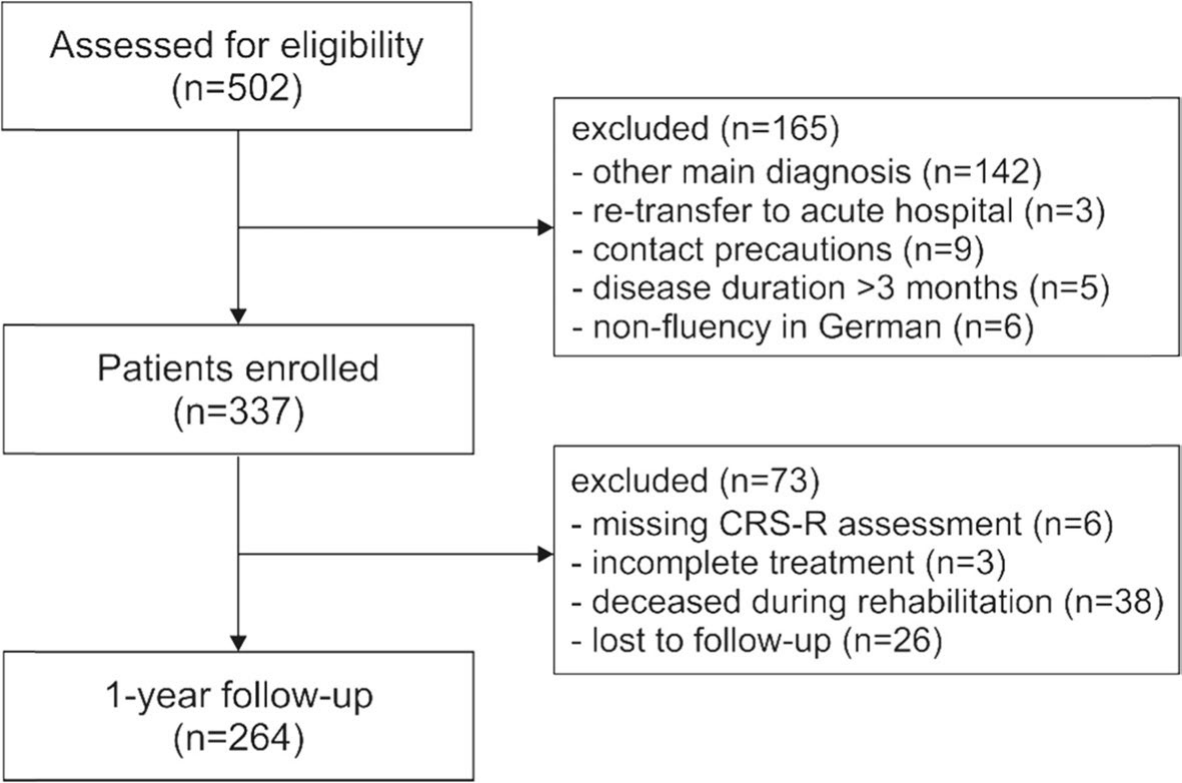
Study flow-chart. Note. CRS-R = Coma Recovery Scale-Revised

The study has been conducted at a neurological rehabilitation center (BDH-Clinic Hessisch Oldendorf, Germany). All patients consecutively admitted to intensive or intermediate care units and discharged between June 2018 and May 2020 were screened for eligibility (*N* = 502, see Fig. 1). Patients were included in the study, when they were (i) at least 18 years old and (ii) suffering from stroke, intracranial hemorrhage, traumatic brain injury or hypoxic brain damage. Patients with other central or peripheral nervous system disorders (*n* = 142), retransfers to an acute-care hospital within the first 3 days (*n* = 3), contact precautions due to colonization with multi-drug resistant bacteria (*n* = 9), disease durations beyond 3 months (*n* = 5) and non-fluency in German (*n* = 6) were excluded from the study.

In Germany, neurological rehabilitation comprises six phases [13]: acute care in a hospital (phase A), early neurological rehabilitation (phase B), subsequent rehabilitation (phases C and D), occupational rehabilitation (phase E) and long-term care (phase F, in specialized nursing facilities). The study focuses on the outcome of early neurological rehabilitation (phase B), refers to the post-acute, multimodal treatment of severely impaired patients. Patients are transferred to subsequent rehabilitation phases when they are able to actively participate in therapies lasting 30 min or longer at least twice daily.

### Data collection

Demographical and clinical data including age, sex, etiology, time since injury, functional status, consciousness, length of stay and type of discharge (e.g., nursing care, returning home) were collected during post-acute rehabilitation. One year after the patient was discharged from post-acute rehabilitation, the outcome was assessed with the Glasgow Outcome Scale-Extended (GOSE) [14].

#### Functional status

The functional status was assessed upon admission using the Barthel Index (BI) [15]. The BI measures functional independence in the activities of daily life through a panel of ten ordinal-scaled items resulting in a scale of 0 to 100 (with 0 being completely dependent and 100 being completely independent). It was rated once a week as part of regular clinical care by a team of nurses, therapists, and physicians.

#### Consciousness

The German version of the Coma Recovery Scale-Revised (CRS-R) scale [16] was used to assess the responsiveness and to quantify the level of consciousness in each patient. The CRS-R scale consists of 23 hierarchically organized items divided into five functional subscales (auditory, visual, motor, oromotor/verbal, communication) and an arousal scale. In outcome studies on prolonged disorders of consciousness, sometimes the total CRS-R score is used as well [7, 8]. Despite the fact that this sum of subscale values, ranging between 0 and 23, does not reflect a continuous variable (e.g. a ‘2’ on the visual subscale reflects a sign of consciousness, while a ‘2’ on the auditory subscale does not) we decided to calculate these sum scores in order to allow for interstudy comparisons.

UWS is diagnosed when patients show either reflexive responses such as visual or auditory startle, localization of sounds, flexion withdrawal, abnormal posturing, oral reflexive movements, or no response. In order to classify MCS, there must be clear evidence of at least one of the following signs: consistent or reproducible movement to command, recognition or localization of objects, visual pursuit, fixation, automatic motor response, object manipulation, localization of noxious stimuli, intelligible verbalization, and nonfunctional intentional communication. Functional communication and/or functional object use indicate the emergence from MCS (eMCS). The first CRS-R assessment was conducted 3 days after admission to inpatient rehabilitation. Subsequently, weekly follow-up examinations during the first month and a final examination at the end of early rehabilitation were performed. For the study, values upon admission and at discharge were used.

#### One-year outcome

For the one-year follow-up, the outcome of surviving patients was assessed with the GOSE [14]. Therefore, individuals who have been specified as a caregiver during inpatient rehabilitation were contacted by phone. When the phone number was incorrect or the caregiver did not respond, an internet search was conducted to obtain further (contact) information. In a few cases, professional care facilities or professional guardians were contacted. The GOSE measures the outcome of brain injuries after discharge from inpatient treatment using an eight-point scale (1 = death; 2 = vegetative state, 3 = lower severe disability, 4 = upper severe disability, 5 = lower moderate disability, 6 = upper moderate disability, 7 = lower good recovery, 8 = upper good recovery). To determine the outcome category for each patient, the structured interview proposed by Wilson and colleagues [17] has been conducted.

### Statistical analyses

Statistical Analyses were conducted using SPSS version 26. Differences were considered significant at *p* < .05. Descriptive statistics are presented as median and interquartile range ([IQR], 25th and 75th percentiles) or numbers and percentages, depending on the type of variable. Chi^2^ tests and Mann-Whitney U tests were conducted to compare group differences. Linear relationships were examined with the Spearman correlation coefficient.

The Kaplan-Meier method and log-rank test were used to estimate the cumulative probability of survival at follow-up, stratified for the level of consciousness upon admission to post-acute rehabilitation. To further evaluate, which factors contribute to one-year mortality, a univariate Cox’s proportional hazards model was used. Significant associations are presented as hazards ratios (HR) with their corresponding 95% confidence intervals (CI).

A multivariate binary logistic regression analysis was performed to predict the outcome at follow up. The Glasgow Outcome Scale-Extended was defined as the dependent variable, which was dichotomized into favorable (GOSE≥4) and unfavorable (GOSE< 4) outcome. Age, sex, etiology, time since injury, length of stay, functional status and consciousness were entered as independent variables. For the model, odds ratios (OR) including 95%-CI and explained variance (Nagelkerke’s R^2^) are reported. The goodness of fit of the model was assessed with the Hosmer and Lemeshow test for logistic regression.

## Results

### Patients

Of the 502 patients screened for eligibility, 337 patients (67.1%) met the inclusion criteria. Among these, 73 (21.7%) had to be excluded from further analyses (see Fig. 1 for reasons). Finally, data of 264 patients were analyzed, including 174 males (65.9%) and 90 females (34.1%). The median age of the study population was 62 years (IQR = 51–75). The cause of brain damage was non-traumatic in *n* = 183 patients (69.3%) and traumatic in *n* = 81 (30.7%). Non-traumatic diagnoses included stroke (*n* = 78, 29.5%), intracranial hemorrhage (*n* = 84, 31.8%), and hypoxic brain damage (*n* = 21, 8.0%). Detailed patient characteristics are presented in Table 1.

**Table 1:** Patient characteristics available during post-acute rehabilitation presented for all patients (*n* = 264) and the two outcome groups (favorable vs. unfavorable)

	Total (*n* = 264)	Favorable outcome (*n* = 63)	Unfavorable outcome (*n* = 201)
Upon admission			
Age at event (years)	62 (51–75)	56 (44–69)	64 (53–77)
Male/Female (n)	174/90	127/74	47/16
Time since injury (days)	18 (12–28)	16 (10–21)	20 (13–29)
Do-not-resuscitate order (n)	39	2	37
Barthel Index	10 (10–15)	10 (10–15)	10 (10–10)
CRS-R	10 (4–16)	14 (11–23)	8 (3–14)
Level of consciousness			
*UWS*	99 (37.5%)	3 (4.8%)	96 (47.8%)
*MCS*	100 (37.9%)	32 (50.8%)	68 (33.8%)
*eMCS*	65 (24.6%)	28 (44.4%)	37 (18.4%)
At discharge			
Length of stay (days)	87 (53–112)	41 (28–69)	98 (68–115)
Barthel Index	20 (15–35)	35 (30–70)	15 (15–25)
CRS-R	22 (11–23)	18 (8–23)	23 (23–23)
Level of consciousness			
*UWS*	44 (16.7%)	–	44 (21.9%)
*MCS*	56 (21.2%)	1 (1.6%)	55 (27.4%)
*eMCS*	164 (62.1%)	62 (98.4%)	102 (50.7%)
Type of discharge			
*Professional care facility*	125 (47.3%)	3 (4.8%)	122 (60.7%)
*Subsequent rehabilitation phase*	96 (36.4%)	57 (90.5%)	39 (19.4%)
*Home care*	25 (9.5%)	2 (3.2%)	23 (11.4%)
*Transfer to other facility*	18 (6.8%)	1 (1.6%)	17 (8.5%)

*Note*. Values are frequencies (sex, level of consciousness, type of discharge) or medians and interquartile ranges (all other variables). *CRS-R*= Coma Recovery Scale-Revised; *eMCS* = emergence from Minimally Conscious State; *MCS* = Minimally Conscious State; *UWS*=Unresponsive Wakefulness Syndrome

**Fig. 2:**
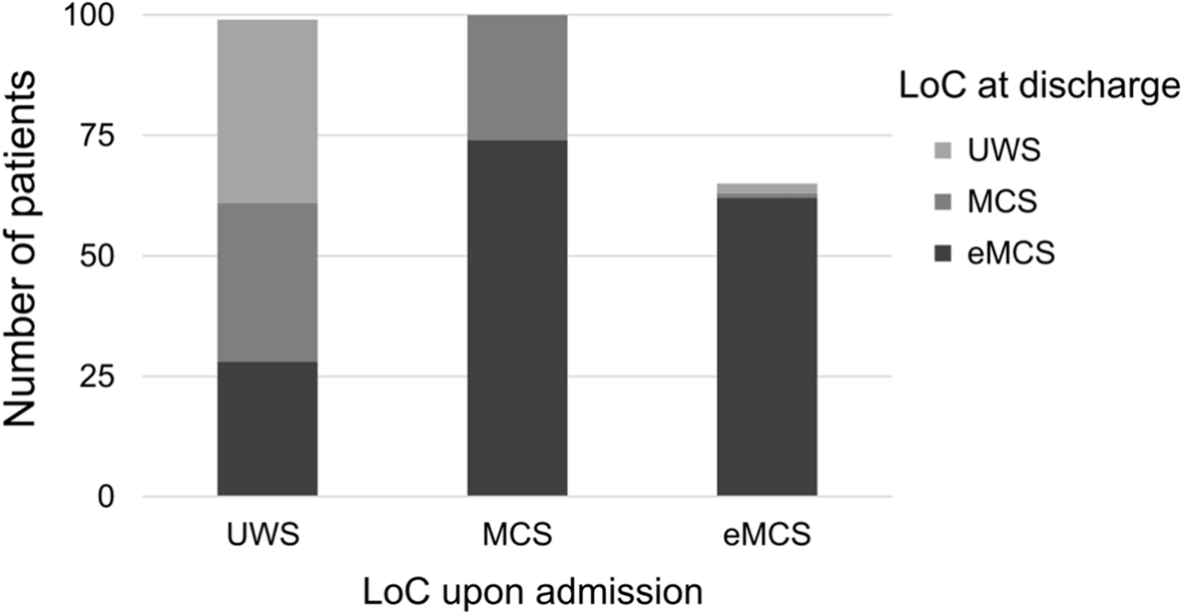
Level of consciousness upon admission and at discharge from early neurological rehabilitation. Note. eMCS = emergence from Minimally Conscious State; LoC = Level of Consciousness; MCS = Minimally Conscious State; UWS=Unresponsive Wakefulness Syndrome

Figure 2 illustrates the changes in the level of consciousness obtained in the first and the final CRS-R assessment. The first CRS-R assessment classified 99 patients as UWS (37.5%), 100 patients as MCS (37.9%) and 65 patients (24.6%) were fully conscious (hereafter termed as “eMCS”). At the end of post-acute rehabilitation, 61 UWS patients (61.6%) had recovered consciousness, with 33 patients (33.3%) transitioning to MCS and 28 patients (28.3%) to eMCS. Among MCS, 74 patients (74%) showed functional recovery to eMCS. Most eMCS patients (95.4%) were re-classified as eMCS in the final CRS-R assessment, indicating the persistence of this level of consciousness. Altogether, 135 patients (51.1%) improved in consciousness. At this point it is important to note, that 65 patients (24.6%) already scored at the ceiling in the first CRS-R assessment and were therefore fully conscious.

### Outcome one year after discharge

The follow-up interviews were conducted 360 to 375 days after discharge from post-acute rehabilitation. Most interviews were performed either with close relatives of the patient (spouse: *n* = 98, 37.1%; children: *n* = 63, 23.9%; siblings: *n* = 19, 7.2%; parents: *n* = 16, 6.1%) or the patient (*n* = 22, 8.3%). In some cases, staff of the current nursing home or professional caregivers were contacted, or an internet search showed obituaries (*n* = 46, 17.4%). Results of the interviews are presented in Table 2. The majority of patients were either dead (*n* = 71; 26.9%), in a vegetative state (*n* = 28; 10.6%) or had a severe disability (*n* = 124; 47.0%), whereas only few patients showed a moderate disability (*n* = 18; 6.8%) or a good recovery (*n* = 23; 8.7%) 1 year after post-acute rehabilitation.

**Table 2:** Number of patients in each category of the Glasgow Outcome Scale-Extended at one-year follow-up

Outcome category		n (%)
Death		71 (26.9)
Vegetative state		28 (10.6)
Severe disability	*lower*	102 (38.6)
	*upper*	22 (8.3)
Moderate disability	*lower*	12 (4.5)
	*upper*	6 (2.3)
Good recovery	*low*	5 (1.9)
	*upper*	18 (6.8)

**Fig. 3:**
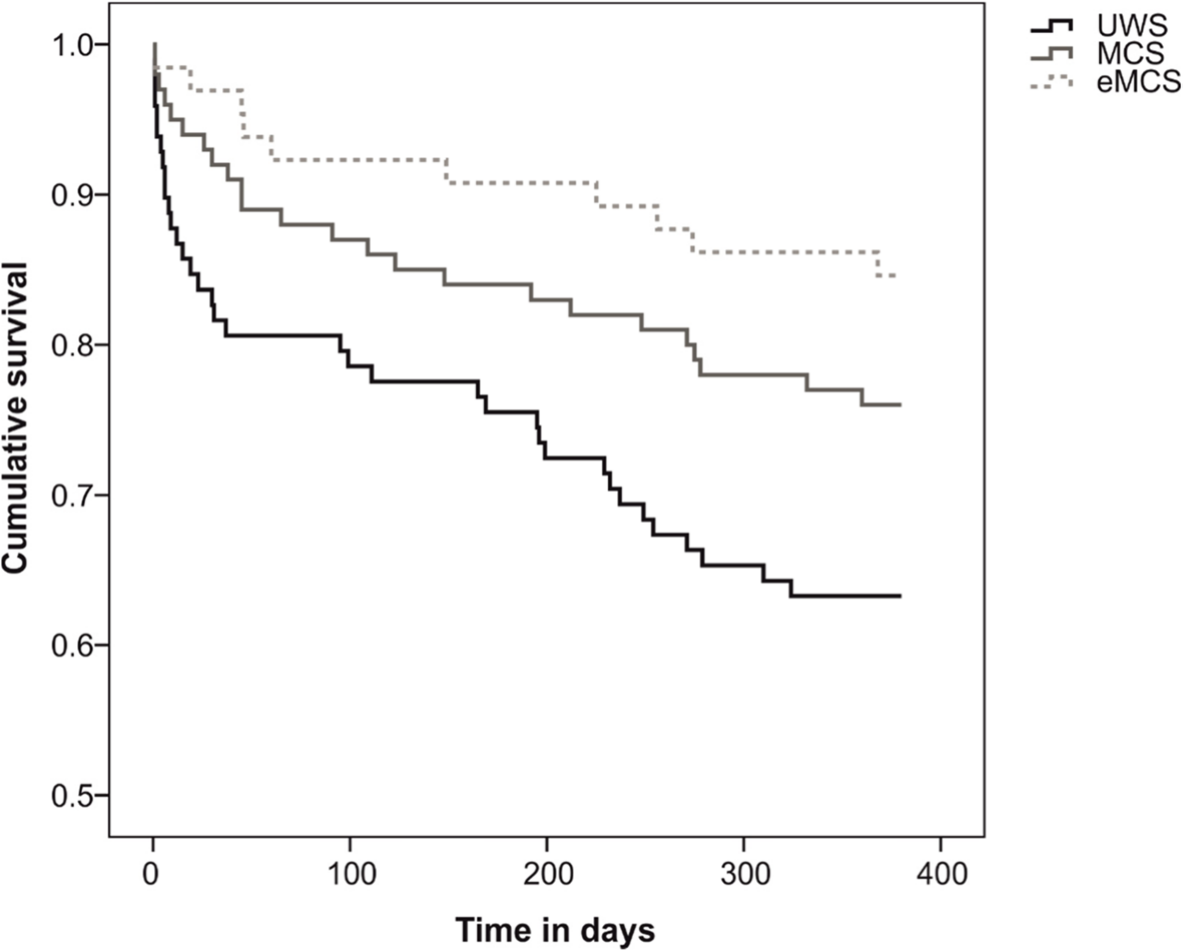
Kaplan-Meier curves for the cumulative probability of survival at follow-up, stratified for the level of consciousness. Note. eMCS = emergence from Minimally Conscious State; MCS = Minimally Conscious State; UWS=Unresponsive Wakefulness Syndrome

The 71 deaths (26.9%) occurred after a median time of 217 days (IQR = 123–349). Causes of death included sudden cardiac arrest (*n* = 12), further stroke (*n* = 3), traumatic brain injury after fall (*n* = 2), and other complications (e.g., ileus, pneumonia, fever, influenza infection, internal bleeding, inflammation, *n* = 22). Two relatives indicated that the patients were treated palliatively. For the other patients (*n* = 30), the cause of death could not be determined, either because the professional guardians or relatives were not able to provide any information, or the information was taken from obituaries. Deceased patients were older (Z = -4.888; *p* < .001) and had lower CRS-R scores upon admission (Z = -3.185; *p* < .01) and at discharge (Z = -4.501; *p* < .001) compared to survivors. Survival rates differed between patients being UWS (*n* = 37; 37.4%), MCS (*n* = 24; 24.0%) or eMCS (*n* = 10; 15.4%) upon admission (Chi2 = 10.335; *p* < .01). The Kaplan-Meier method revealed that the cumulative survival during the first year after discharge was significantly worse in UWS compared to MCS (log rank = 4.099; *p* < .05) and eMCS (log rank = 8.978; *p* < .01), see Fig. 3.

The proportion of deaths did not differ as a function of etiology, as there were no differences in mortality between non-traumatic (*n* = 51; 27.9%) and traumatic (*n* = 20; 24.7%) cases (Chi2 = 0.288; *p* = .591). In a Cox proportional hazards model with death (yes/no) as the dependent variable, age (HR = 1.056; CI = 1.034–1.078, *p* < .001) and discharge CRS-R score (HR = 0.949; CI = 0.912–0.987, *p* < .01) turned out to have a significant effect.

**Fig. 4:**
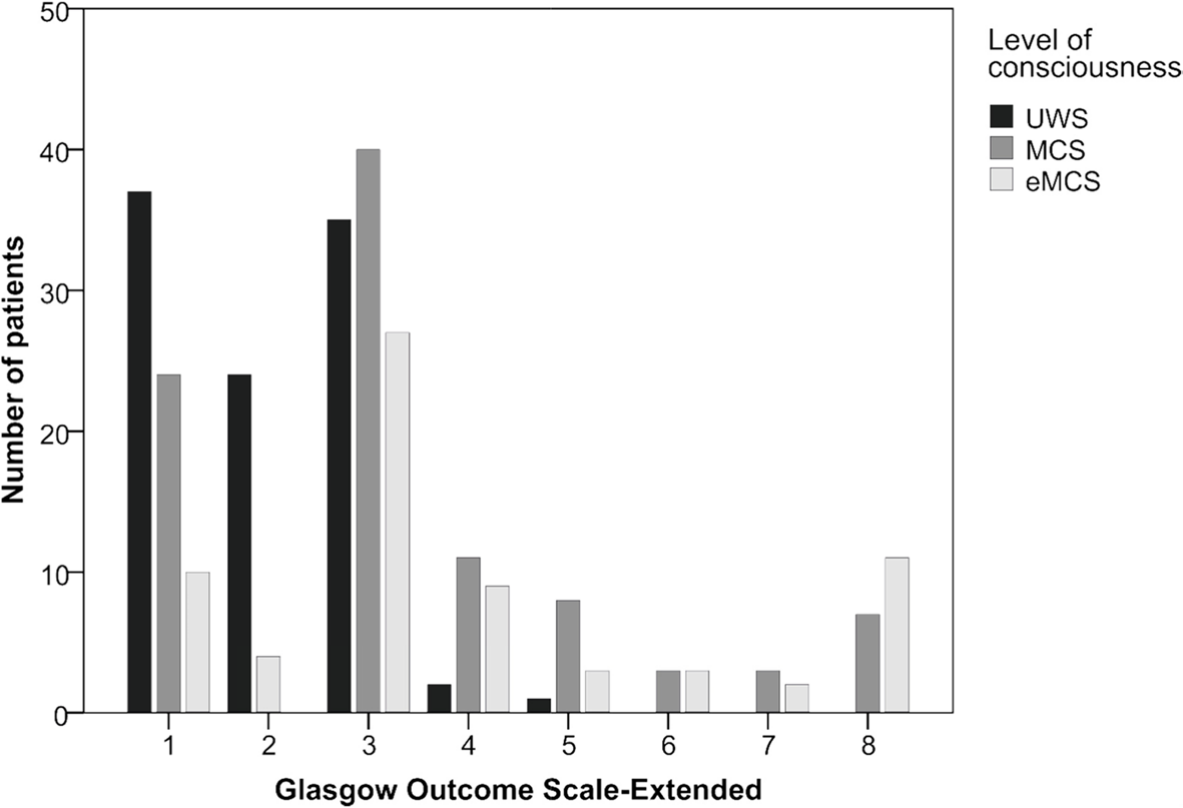
Results of the Glasgow Outcome Scale-Extended, stratified for patient’s level of consciousness upon admission to post-acute rehabilitation. Note. GOSE = Glasgow Outcome Scale-Extended; eMCS = emergence from Minimally Conscious State; MCS = Minimally Conscious State; UWS=Unresponsive Wakefulness Syndrome

Individual results of the Glasgow Outcome Scale-Extended, stratified for different levels of consciousness upon admission, are presented in Fig. 4. Progress to moderate disability was observed in one UWS patient only, in contrast to patients who showed signs of minimal consciousness upon admission. In this group, at least 21% of patients showed moderate to good recovery, allowing them to resume almost all activities of daily living (moderate disability: *n* = 11; 11%) or even normal occupational and social activities (good recovery: *n* = 10; 10%).

The GOSE score 1 year after discharge correlated with the CRS-R total score upon admission (*r* = 0.429; *p* < .001) and at discharge (*r* = 0.528; *p* < .001). At follow-up, 201 patients (76.1%) had an unfavorable outcome. Univariate analyses revealed that these patients were older (Z = -3.581; *p* < .001) and stayed longer in the acute-care hospital (Z = -2.839; *p* < .01) than patients with a favorable outcome. This finding might be explained by a more severe impairment upon admission, as indicated by a lower BI (Z = -3.018; p < .01) and CRS-R score (Z = -6.395; *p* < .001). Moreover, patients with an unfavorable outcome stayed longer in post-acute rehabilitation (Z = -7.510; *p* < .001) and showed fewer improvements in BI (Z = -8.298; *p* < .001). The proportion of favorable outcome differed as a function of the level of consciousness upon admission (Chi2 = 40.500; *p* < .001). A favorable outcome was found in 3 (3.0%), 32 (32.0%) and 28 (43.1%) patients classified as UWS, MCS and eMCS, respectively. Furthermore, the outcome was worse for non-traumatic compared to traumatic brain injuries (Chi2 = 11.161; *p* < .01).

Using binary logistic regression, unfavorable outcome at follow-up was predicted by age (OR = 0.95; CI = 0.92–0.98; *p* < .001), non-traumatic brain damage (OR = 2.81; CI = 1.10–7.19; *p* = <.05), discharge CRS-R score (OR = 1.28; CI = 1.06–1.55; *p* = <.05), and length of stay (OR = 0.98; CI = 0.97–0.99; *p* < .001). Overall, these predictors accounted for 62% of the total variance of the outcome parameter (Nagelkerke’s R^2^ = 0.618). The Hosmer and Lemeshow test was not significant (Chi^2^ = 3.158; *p* = .924), confirming goodness-of-fit for the model tested.

## Discussion

This study aimed to investigate the outcome of critically ill patients 1 year after post-acute neurological rehabilitation. At follow-up, most patients had an unfavorable outcome, which was independently predicted by age, non-traumatic etiology, length of stay and discharge CRS-R total score. The overall mortality rate at follow-up was 27%. Deceased patients were older and more frequently in UWS upon admission to post-acute rehabilitation. Age and the CRS-R score at discharge proved to be independent predictors for mortality in a Cox regression analysis.

Age was an independent predictor for both survival and functional outcome 1 year after discharge from post-acute rehabilitation, with younger patients being more likely to have a favorable outcome. Age has repeatedly been identified as an outcome predictor in critically ill patients [10, 18–20]. The relationship between increased mortality and poorer outcome among elderly patients is probably explained by multiple factors. A possible explanation might be that elderly patients exhibit higher morbidity and altered brain plasticity, which may influence their ability to recover after injury [21, 22]. Etiology did not affect the mortality rate, but the functional outcome was worse for non-traumatic etiologies compared to traumatic etiologies. This result is in line with previous studies showing that traumatic etiologies are associated with better outcome than non-traumatic etiologies [2, 10, 18, 23].

Results of the study further revealed that the outcome at follow-up was associated with consciousness. This was reflected by the fact that the CRS-R score at discharge was an independent predictor for both mortality and functional outcome 1 year after discharge. On the other hand, patients being MCS upon admission showed a better outcome than UWS patients. These results confirm previous findings that MCS patients are more likely to recover within the first year after disease onset than patients in UWS, in particular when they improve to MCS within the first month [2, 5]. While a previous study from our group has shown that initial CRS-R score as well gains after 4 weeks predicted outcome at the end of post-acute rehabilitation [12], these findings do not apply to the one-year outcome. Since a considerable number of patients regain minimal or full consciousness during post-acute rehabilitation, the final assessment of consciousness may have a higher predictive value for the long-term outcome than the initial assessment.

Although some of these critically ill patients showed improvements in consciousness 1 year after discharge, most of them stayed severely impaired. These patients are partially or totally dependent on nursing care during activities of daily living. Their ability to participate in most of their previous personal, social, and occupational activities is markedly reduced. This finding might be regarded as a devastating result, questioning the efficacy of post-acute rehabilitation. However, it has been shown in previous studies that neurological rehabilitation is effective in reducing the dependence on nursing care, quality of life [24] and the weaning of neurological patients from mechanical ventilation [25, 26]. The present study used a relatively rough outcome assessment, which is not suitable to reveal subtle but relevant improvements. Moreover, the GOSE does not provide any information about the quality of life of the patients. It is conceivable that some of them are grateful and satisfied although they are functionally dependent on nursing care, while other patients in the same category struggle with their situation and feel disappointed and angry. Therefore, the perceived quality of life and individual coping mechanisms should be assessed in future studies, as well.

In addition, only patients admitted to intensive or intermediate care units were enrolled, although post-acute neurological rehabilitation includes patients admitted to peripheral wards, too. Including these patients who represent a significant proportion of post-acute neurological rehabilitation patients, may have yielded more favorable outcomes. Thus, the findings of the present study do not allow a general evaluation of post-acute neurological rehabilitation.

## Conclusion

Age proved to be an independent predictor of mortality and outcome 1 year after discharge from post-acute rehabilitation. This might be due to altered brain plasticity and morbidity. The present study further revealed that the discharge CRS-R score may be more relevant to predict long-term outcome than the initial assessment upon admission. Since a considerable number of patients show improvements in consciousness during post-acute rehabilitation, it seems plausible that the final assessment of consciousness has a higher predictive value for the long-term outcome than the initial one.

### Limitations

There are some limitations that have to be addressed. Patients classified as eMCS in the first CRS-R assessment might previously have been either minimally or fully conscious. Thus, the term “eMCS” might not be suitable for all patients. Moreover, this group is usually excluded from studies to compare the outcome of UWS and MCS patients. However, the present study aimed to investigate the outcome of all critically ill patients submitted to intensive care or intermediate care units of a post-acute rehabilitation facility. Another limitation is the one-time assessment of the level of consciousness at study entry and at discharge. A recent study suggests that at least five separate CRS-R assessments over 2 weeks might be necessary to establish an accurate diagnosis [27].

The one-year outcome was assessed with the Glasgow Outcome Scale-Extended, which does not allow a distinction between UWS and MCS. Such a distinction would have enabled more detailed information about the recovery of consciousness (i.e., UWS patients who progressed to MCS) and functional recovery (i.e., UWS/MCS patients who progressed to eMCS) at one-year follow-up. Future studies should therefore pose questions in the interview allowing to classify the current level of consciousness. In addition, the research associate conducting the GOSE interviews was also involved in collecting the CRS-R data 1 year before and was therefore not completely blinded to the diagnoses and the outcomes of all patients.

It should also be noted that the German model of neurological rehabilitation is quite different from other countries since some patients entering post-acute rehabilitation are still comatose and mechanically ventilated. In other countries, these patients might not be eligible to enter rehabilitation and would rather stay in an ICU of an acute-care hospital. In addition, post-acute rehabilitation is offered for all kinds of neurological and neurosurgical disorders (anoxic, traumatic, vascular and other injuries) “under one roof” instead of even more specialized centers. These differences might limit the transferability of our results to other countries with different healthcare systems.

## Data Availability

The datasets supporting the conclusions of this article are available from the corresponding author on reasonable request.

## Abbreviations

BI: Barthel Index
CI: Confidence interval
CRS-R: Coma Recovery Scale-Revised
eMCS: Emergence from MCS
GOSE: Glasgow Outcome Scale-Extended
HR: Hazards ratio
IQR: Interquartile range
MCS: Minimally conscious state
OR: Odds ratio
UWS: Unresponsive wakefulness syndrome
